# Adherence to iron folate supplementation and associated factors among pregnant women attending antenatal care at public hospitals in Jigjiga Town, Somali Region, Ethiopia 2020

**DOI:** 10.11604/pamj.2021.40.196.27958

**Published:** 2021-12-01

**Authors:** Neima Ridwan, Abdulfeta Shafi

**Affiliations:** 1 Department of Midwifery, School of Medicine, Jigjiga University, Jijiga, Ethiopia,; 2 Department of Statistics, Worabe University, Worabe, Ethiopia

**Keywords:** Adherence, iron folate supplementation, pregnancy, anemia

## Abstract

**Introduction:**

pregnant women are at particular risk of folate and iron deficiency due to their increased requirements, which can be difficult to meet through diet alone. Poor adherence to the supplement makes the prevalence of anemia among pregnant women high, which is associated with increased maternal and perinatal morbidity and mortality. The objective of study was to assess magnitude of adherence to iron-folic supplements and their associated factors among pregnant women attending antenatal care (ANC) at public hospitals in the Jigjiga Town, Somali Region.

**Methods:**

an institutional based cross-sectional study was conducted on 290 pregnant during antenatal care service in two public hospitals. Mothers were randomly selected and interviewed using questionnaires. Multiple logistic regressions used to show the effect of independent variables.

**Results:**

in this study, 290 women interviewed and the response rate was 91%. Nearly 54.9% were adherent to iron folic supplementation. The odds of adherence for secondary and above educated woman was almost three times (adjusted odds ratio [AOR]: 2.843; 95% CI: 1.177-6.865; P=0.020) that of illiterate. The odds of adherence for more than four visit women is almost twice of less than four visits (AOR: 1.991; 95% CI: 1.098-3.610; p=0.023). Similarly, odds of adherence for knowledgeable woman about iron folate supplement were twice of ill-informed women (AOR: 2.090; 95% CI: 1.134-3.852; P=0.018).

**Conclusion:**

adherence to iron folic supplementation was very poor. Number of ANC visits, education of women, knowledge about iron folic supplement, history of still birth, and age were significant determinants to adherence.

## Introduction

Pregnancy is one of the more important periods in life when increased micronutrients, and macronutrients are most needed by the body; both for the health and well-being of the mother and for the growing fetus and newborn child [1]. Pregnant women are at particular risk of folate and iron deficiency due to their increased requirements, which can be difficult to meet through diet alone [2]. Anemia is one of iron deficiency disease. It is a condition in which the number of red blood cells or their oxygen-carrying capacity is insufficient to meet physiologic needs, which vary by age, sex, altitude, smoking, and pregnancy status [3]. Women become anemic during pregnancy because of rapid tissue growth and increasing fetal needs [4]. It is estimated that the blood volume increases approximately 50 percent during pregnancy, although the plasma amount is disproportionately greater. This causes dilation of the blood, making the hemoglobin concentration fall, which results in anemia [5].

Currently, World Health Organization (WHO) recommendations for the prevention, control, and treatment of anemia in pregnant women daily oral iron and folic acid supplementation with 30 mg to 60 mg of elemental iron and 400 µg (0.4 mg) folic acid as part of antenatal care, to reduce the risk of low birth weight, maternal anemia and iron deficiency [6]. In addition to iron and folic acid, supplements may be formulated to include other vitamins and minerals, according to the United Nations Multiple Micronutrient Preparation (UNMAP), to overcome other possible maternal micronutrient deficiencies.

Adherence to therapies is a primary determinant of treatment success. Poor adherence attenuates optimum clinical benefits and therefore reduces the overall effectiveness of health systems [7]. In Ethiopia, anemia is a moderate public health problem [8]. One quarter of reproductive aged woman are anemic in Ethiopia [9]. One of strategies to control problem associated with iron deficiency anemia (IDA) and its adverse health consequences on pregnant women and their neonate is provision of iron folate supplementation during pregnancy, meanwhile compliance to the supplementation remain low.

According to Ethiopian mini demographic health survey report [9], the compliance to iron folate supplementation is very poor. About 89% of women didn´t take recommended numbers of iron tablets during their last pregnancy. This survey report also shows that about 60% took less than 60 days, and less than 11% took for 90 days or more during their last pregnancy. There was regional disparity among women who take IFA tablets for 90+ days, ranging from a low 1.8% in Somali Region to 22% in Dire Dawa. This mini report shows 81.2% of pregnant women in Somali regional state didn´t take iron folic acid supplement tablets during their last pregnancy. Women in the Somali and Afar Regions are most highly affected by anemia, with rates of 60% and 45%, respectively [9]. Even though, the problem is common to best of researchers´ knowledge, there is no study done on the level of adherence to iron folate supplementation among pregnant women as well as associated factor in the Jigjiga Town. Therefore, identifying the level of adherence and its associated factors in pregnant women would be essential for evidence based intervention. Therefore, this study aimed to assess magnitude of adherence to iron folic supplementation (IFS) and its associated factors among pregnant women attending antenatal care (ANC) at two public hospitals in Jigjiga Town.

### Operational definition

**Adherence:** pregnant women are said to be “adherent” to iron folate supplement, if they took 65% or more of the supplement, equivalent to taking the supplement at least 4 days a week during one month period using recording, reporting and checking their cards [10,11].

**Knowledge about anemia:** was determined by five questions. Summing up all correct responses provided by women and those who scores mean and above value considered as knowledgeable about anemia.

## Methods

**Study area and period:** this study was conducted in two public hospitals namely Karamara General Hospital and Shekh Hassen Yebere Referral Hospital in Jigjiga Town, which is located in Somali Regional State, eastern part of Ethiopia. Data collection was done from March 1^st^-30^th^ 2020.

**Study design, population and sampling methods:** institution based cross-sectional study design was conducted. All pregnant women who attend ANC services during data collection, have at least one ANC visit before, and supplemented with iron folate for current pregnancy at two public hospitals were included. Total of 290 pregnant women were randomly selected and interviewed.

**Data collection tool and producers:** face to face interview questionnaire was used to collect data from pregnant women. The questionnaire was developed by reviewing different literatures. Internal consistency was checked by Cronbach´s alpha and the items had good internal correlation (Cronbach´s alpha=0.6). Sample size was proportionally allocated to hospitals. About 162 study participants were interviewed from Shek Hassein Yaber Referral Hospital and 128 from Karamara Hospital. Mother randomly selected and interviewed until desired sample size obtained in specified study period by two-trained midwiferies.

**Sample size:** the sample size for this study was determined using single population proportion assuming that expected adherence rate for iron folic supplement is 22% (p=0.22) from afar region (α 5%, margin of error (d=5%) and a non-response rate of 10%) [12].


$$n=\frac{{\left(Z_{\frac{\alpha }{2}}\right)}^{2}p.q}{d^{2}}$$

where p=proportion of adherence rate, q=proportion of non-adherence rate, α=level of significance, zα./2=1.96, standardized value from Z-table based on α equals to 5%, finally, adding 10% non-response rate n=290.

**Inclusion criteria and exclusion criteria:** all pregnant women who attend ANC service during data collection and have at least one ANC visit before and supplemented with iron folate for one month for current pregnancy before the date of interview were included. However, pregnant women with mental disorder, unable to hear and/or speak, anemic, women attending ANC for the first time and very sick were excluded from the study.

**Study variable**

**Dependent variable:** adherence to iron folate supplementation. Mother who had taken four or more iron folate supplements per week for immediately preceding 1 month.

**Independent variable:** includes socio-demographic factors, pregnancy related, and knowledge about supplement, anemia and health care service related factor.

**Data processing and analysis:** data were entered into a computer by SPSS version 20 for analysis. The participants missed information more than 5% was excluded from the analysis and for less than 5% multiple imputations was used to substitute missing value. Descriptive statistics like frequency, proportion, standard deviation, and mean of variables were calculated. Univariate logistic regression was done to see the association of each independent variable with the outcome variables and those predictor variables which have a p-value ≤ of 0.25 were entered into multiple logistic regression models to identify the effect of each explanatory variable. Finally, the model constructed by including all predictors into multiple logistic regression models to identify the effect of each explanatory variable on adherence rate. So that, adjusted odds ratio or CI for exp (B) was used to interpret the effect of each variable, and statistical significance was declared at P-value <0.05. The goodness of fit for multiple logistic regression models assessed using Hosmer and Lemeshow methods.

**Ethical approval and participant consent:** ethical approval was obtained from the institutional review board of the University of Jigjiga and an official letter was submitted to the public health institutions. The collected data used for this research purpose only and kept with complete confidentiality. Verbal informed consent was obtained from the study participants and personal identifiers were excluded during the data collection to assure confidentiality.

## Results

**Participant:** two hundred ninety (290) pregnant women who attended ANC were interviewed in this study and the response rate was 91%. The mean ± (SD) age of respondents were 26.8 (± 5.48) years. About one third of women 89 (33.7%), were in the age group of 26-30 years and more than 214 (81%) respondents were less than 30 years. Most of mothers 207 (78.4%) were Muslim and 80.7% of total participants were urban residence.

**Socio-demographic characteristics of respondents:** the mean ± standard deviation (SD) age of respondents was 26.8 ± 5.48 years. About one third of the study participants (33.7%), were in the age group of 26-30 years and more than 81% respondents were less than 30 years. Most of the respondents (78.4%) were Muslim and 80.7% of total participants were urban residence. Greatest proportions 97.7% (258) of the respondents were married. More than one-third 35.6% (95) of the study participants´ family size was in the range of 0-3. Table 1 shown below summarizes socio demographic status of pregnant women who participated in the study.

**Table 1:** socio-demographic characteristics of respondents

Variable	Variable's categories	Frequency	Percentage
Age	<20	43	15.5%
	21-25	84	31.8%
	26-30	87	33.7%
	31-35	36	13.3%
	≥36	14	5.7%
Marital status	Married	258	97.7%
	Single	6	2.3%
Religion	Muslim	207	78.4%
	Orthodox	46	17.4%
	Protestant	9	3.4%
	Other	2	0.8%
Educational status of respondent	Illiterate	107	40.5%
	Informal education	22	8.30%
	Primary education	56	21.2%
	Secondary education	32	12.1%
	College and above	47	17.8%
Residence	Urban	213	80.7%
	Rural	51	19.3%
Occupational status of respondent	Homemaker	193	72.7%
	Merchant	24	9.1%
	Government employer	33	12.9%
	Private employer	11	4.2%
	Laborer	2	0.8%
	Other	1	0.3%
Educational status of husband	Illiterate	36	13.6%
	Informal education	31	11.7%
	Primary education	38	14.4%
	Secondary education	42	15.9%
	College and above	111	42.0%
Occupational status of husband	Pastoralist	23	8.70%
	Government employer	97	36.7%
	Private employer	38	14.4%
	Merchant	63	23.9%
	Laborer	25	9.50%
	Other	18	6.8%

**Figure 1:**
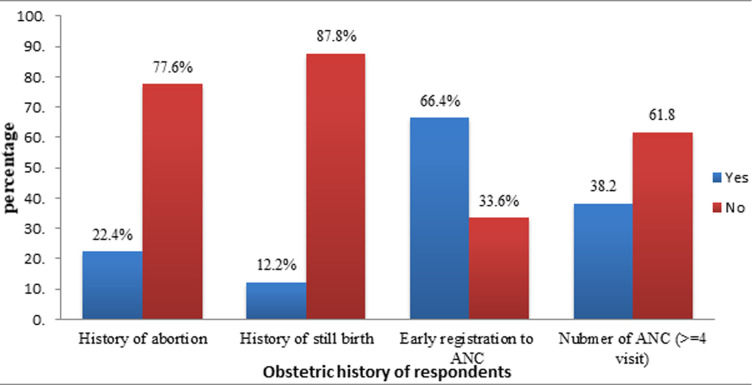
obstetric history of respondents

**Obstetric history of respondents:** regarding obstetric history, the majority of 241 (91.3%) pregnant women were multigravida, and about 175 (66.3%) of the pregnant women enrolled for ANC before 16 weeks of gestational age. Among mothers who started antenatal care follow-up, 32 (12.1%) had more than four antenatal care visits (Figure 1).

**Figure 2:**
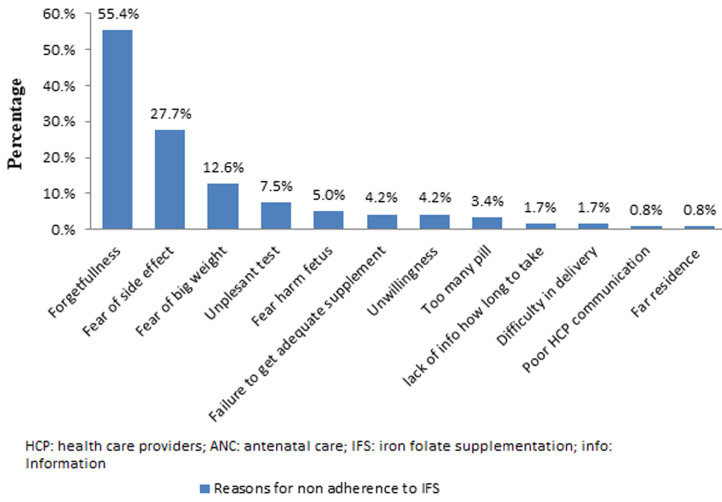
reason for non-adherence to iron folic supplementation

**Knowledge of pregnant women about anemia and iron folate supplementation:** about 177 (67%) pregnant women were knowledgeable about the cause of anemia during pregnancy. Respondents´ knowledge about anemia was determined by summing up all correct responses provided to them. Pregnant women who scored mean and above value were considered as knowledgeable about anemia. Concerning their knowledge about iron folate supplementation during pregnancy, 204 (76.5%) of them were knowledgeable. However, 85 (32.2%) of them had no knowledge about the cause of anemia (Table 2). Common reasons for non-adherence to IFS for participants were forgetfulness 146 (55.4%), fear of side effects 73 (27.7%), and fear of having a big baby 33 (12.6%) took the great proportion (Figure 2).

**Table 2:** knowledge about anemia and iron folate supplement of respondents

Variables	Category	Frequency	Percentage
Cause of anemia	Yes	179	67.8%
	No	85	32.2%
Consequence of anemia	Yes	211	79.9%
	No	53	20.1%
Knowledge about benefit of IFS	Yes	202	76.5%
	No	62	23.5%
Benefit of IFS	Prevent maternal blood lose	116	43.9%
	Decrease infant mortality	101	38.1%
	Prevent birth defect	45	17%
Risk of IFS	Cause big baby	196	74.1%
	Harm fetus	28	10.6%
	Complicate in delivery	12	4.7%
	Other	28	10.6%

IFS: iron folic supplementation

**Adherence to iron and folate supplementation:** based on self-report more than half 145 (54.9%) of pregnant women were adherent to IFA supplementation. That is, among all participants of the study, 54.9% of pregnant women had taken four or more iron folic supplements per week in the previous week preceding the survey. Conversely, 119 (45.1%) of them were non-adherent to iron-folic acid supplementation.

### Multiple logistic regressions

**Factors associated with adherence to iron folate supplementation:** under multiple logistic regression analysis, eight variables that were significant in the univariate logistic regression model were included. The multiple logistic regression fitted model showed that the number of antenatal care services, educational level of mothers', age of mothers', knowledge about iron-folic supplement, and history of stillbirth had a significant effect on the magnitude of adherence. Table 3 summarizes multiple logistic regression output. The odds of adherence for iron folate supplement for women having more than four visits was almost twice of odds of less than four visits (AOR: 1.991; 95% CI: 1.098 - 3.610; P=0.023). Difference for an odd for two categories of visits were significant and pregnant women who had four and more visits were more adhered than less than four visits (Table 3).

**Table 3:** factors associated with adherence to iron folate supplementation

Variables	Category	Adherence to IFS		AOR(95% CI)	P-value
		**Yes**	**No**		
Education status women	Illiterate	50(46.7%)	57(53.3%)		
	Till secondary	59(53.6%)	51(46.4%)	1.043(.56,1.92)	0.892
	Above secondary	36(76.6%)	11(23.4%)	2.843(1.17,6.86)	0.020*
Age (year)	Age ≤25 years	58(45.7%)	69(54.3%)		
	Age>25 years	87(63.5%)	50(36.5%)	2.163(1.24,3.75)	0.006*
Knowledge about anemia	Good knowledge	130(59.9%)	87(40.1%)	1.8000(0.84,3.88)	0.128
	Poor knowledge	15(31.9%)	32(68.1%)		
Knowledge about IFS	Good knowledge	113(64.6%)	62(35.4%)	2.090(1.13,3.85)	0.018*
	Poor knowledge	32(36%)	57(64%)		
Early registration	Yes	105(60.0%)	70(40.0%)	1.452(0.81, 2.60)	0.21
	No	40(44.9%)	49(55.1%)		
Still birth	No	131(56.7%)	100(43.3%)		
	Yes	14(42.4%)	19(57.6%)	2.452(1.04,5.73)	0.039*
Number of ANC visit	<4 times	91(55.8%)	72(44.2%)		
	≥4 times	54(53.3%)	47(46.5%)	1.991(1.09,3.61)	0.023*

IFS: iron folate supplementation; ANC: antenatal care; AOR: adjusted odds ratio; *significant at alpha=0.05

Adherence rate to iron folate supplementation has direct relation with educational status of women. Odds of adherence for more than secondary educated woman was almost three times (AOR: 2.843; 95% CI: 1.177-6.865; P=0.020) that of illiterate women. Meaning that, odds of adherence for more than secondary educated woman increase more than 180% than uneducated women (Table 3). Regarding the age of pregnant women, mothers greater than 25 years old were more adhered than less than 25 years. The odds ratio of adherence for age greater than 25 years old women to those of age less than 25 years is 2.163. This shows odds of adherence for women greater than 25 years are more than twice of age less than 25 years (AOR: 2.163; 95% CI: 1.246-3.754; P=0.006) and the difference of odds within these two age groups is statistically significant at 0.05 (Table 3).

In this study, another factor that is associated with adherence to IFS was knowledge about iron folate supplements. The odds of adherence for a knowledgeable woman about iron folate is more than twice of unconscious (AOR: 2.090; 95% CI: 1.134-3.852; P=0.018). Moreover, women who had a history of stillbirth were more adhered than women who didn´t had a history of stillbirth. The odds of adherence for a woman who had a history of still birth was more than twice of women who didn´t have a history of stillbirth (AOR: 2.452; 95% CI: 1.048-5.733; P=0.039) (Table 3).

## Discussion

In this study, the magnitude of adherence to iron folate supplement assessed. In both hospitals total of 290 women interviewed and 264 mothers gave full information. From this, more than half (54.9%) of pregnant women were adherent to iron folate supplementation. This result is in line with a study conducted in Eastern Terai of Nepal [13] and Burji Districts, Segen Area People´s Zone, Southern Ethiopia (51.4%) [14]. Finding of this study is greater than the study conducted in Kiambu, Kenya (32.7%) [15] and Pakistan (38.3%) [16]. However, the adherence rate in this study was less than the study done in South India (64.7%) [17].

This study revealed that maternal education had a significant association with adherence to iron and folic acid supplementation. The odds of more than a secondary educated woman was almost three times their counter wise. The result supported by other studies done in Nepal [13] and studies done in Addis Ababa, Ethiopia [18], and Mecha District, Western Amhara [19] revealed that maternal education had a significant association with IFS adherence. Educated women may appreciate the benefits gained by complying with iron folate supplements, and have better health-seeking behavior than uneducated mothers´ have.

Woman with four and more ANC service visits were more adhered than woman having less than four ANC service visits. An odd of adherence for iron folate supplement for pregnant woman having more than four visits was almost twice of odds of those less than four visits. This finding is consistent with the study done in urban, peri-urban, and rural communities in Southeast Nigeria [20], Northern Ethiopia [21], and a systematic review and meta-analysis done in Ethiopia [22]. Possible reason for this might be due to the fact that women who had more frequent visit have exposure of information which might be more likely to be knowledgeable about the supplement and also health care providers might helped women during their ANC visits by discussing about benefit of IFS, subsequently end-up with the adherence to IFS.

According to the findings of this study, mothers greater than 25 years old were more adhered than less than 25 years. The odds ratio of adherence for age greater than 25 years old to those of less than 25 years is 2.163. This figure shows odds of adherence for greater than 25 years are more than twice that of age less than 25 years. This finding is in line with a study done in 22 sub-Saharan African countries, South India [17] and Northern Tanzania [23]. Older women may know the benefits of iron supplementation to prevent anemia or having experienced iron deficiency-related adverse outcomes. In addition, younger women are more concerned about their health and pregnancy outcomes.

Another factor that had a significant association with IFS adherence was knowledge about IFS. Well-informed women about IFS were more adhered to IFS. The odds of adherence for knowledgeable woman about iron folate supplement were more than twice of ill informed. The finding of this study was supported by studies done in Indonesia [24], Addis Ababa [18], and Southeast Ethiopia [25]. This could be when women knew the benefit of the supplement that decreases maternal and infant mortality and morbidity more likely to have adherence to the iron folate supplement.

**Limitation of study:** the possible limitations of this study were reports of a mother may under or overestimate compliance rates.

## Conclusion

This study revealed that the adherence rate for iron and folic acid supplementation was too poor. Maternal education, number of antenatal care services, age of mothers, history of stillbirth, and knowledge of pregnant women about iron folic supplements were significantly associated with adherence to iron-folic supplementation. Forgetfulness, fear of side effects, and fear of having a big baby were common reasons for non-adherence to iron-folic supplementation.

**Funding:**this research was partially supported by Jigjiga University. The university has no role in this paper other than funding.

### What is known about this topic

- Iron and folic acid supplementation recommended by WHO during pregnancy;
- Poor adherence to iron and folic acid supplementation;
- Iron folic supplement are very important to control anemia during pregnancy.

### What this study adds

- Reason for non-adherence was identified;
- Adherence rate is poor in Somali Region;
- Commonly, forgetfulness and fear of side effects are reasons for non-adherence.
